# Epidemiological Trends in the Form of Childhood Tuberculosis in a Referral Tuberculosis Hospital in Shandong, China

**DOI:** 10.1155/2020/6142567

**Published:** 2020-08-08

**Authors:** Mao-Shui Wang, Jun-Li Wang, Xin-Jie Liu

**Affiliations:** ^1^Department of Pediatrics, Qilu Hospital, Cheeloo College of Medicine, Shandong University, Jinan, Shandong, China; ^2^Department of Lab Medicine, Shandong Provincial Chest Hospital, Cheeloo College of Medicine, Shandong University, Jinan, Shandong, China; ^3^Department of Lab Medicine, The Affiliated Hospital of Youjiang Medical University for Nationalities, Baise, China

## Abstract

**Background:**

In China, the prevalence of tuberculosis (TB) diseases and epidemiological trends in the TB forms among children are still unclear; a retrospective study was conducted aiming to assess it.

**Methods:**

Between January 2007 and September 2020, 1577 consecutive childhood TB patients (aged ≤ 15 years) were included in the study. Data, including demographic information and underlying diseases, were collected from medical records. Then, patients were categorized and reported according to the anatomical site of TB disease. To analyze the epidemiological trends in the proportion of each form of TB disease, a linear-by-linear association was used, and a *P* value of <0.05 was considered to indicate that a significant change had occurred in the proportion of TB disease over the studied period.

**Results:**

During the fourteen-year study period, a total of 1577 children patients were enrolled, including 954 boys (60.5%) and 623 girls (39.5%), with a mean age of 9.26 ± 5.18 years. Among the studied patients, 810 (51.4%) patients have pulmonary TB, 1137 (72.1%) have extrapulmonary TB, 372 (23.6%) have both conditions, and another 765 (48.5%) extrapulmonary cases presented in isolated form. Pleural TB (29.0%) and tuberculous lymphadenitis (23.7%) were the most frequent two forms of childhood TB. In addition, during the past decade, the proportions of pulmonary TB, pleural TB, and tuberculous lymphadenitis showed an increasing trend (all *P* < 0.05). However, no significant trends in the proportions of other forms of TB disease, such as extrapulmonary TB (*P* > 0.05), tuberculous meningitis (*P* > 0.05), endobronchial TB (*P* > 0.05), and disseminated TB (*P* > 0.05), were found.

**Conclusion:**

Our findings suggest that childhood TB is facing new challenges, and the policy should be adjusted timely to fit the real situation.

## 1. Introduction

Childhood tuberculosis (TB) remains a serious worldwide public health threat. According to the WHO global TB report 2019, an estimated 10.0 million (range: 9.0–11.1 million) people fell ill with TB in 2018 globally, and children accounted for 11% [1]. Fortunately, a recent study employing data from the Centers for Disease Control (CDC) found that, in China, from 2009 to 2015, the incidence of pulmonary TB (PTB) in children decreased by over 50% [2]. Similarly, the data extracted from the Global Health Data Exchange showed that from 1990 to 2017, decreasing trends in prevalence (average annual percent change: −0.5%), incidence (−3.2%) of all tuberculosis cases were observed [3].

In contrast to adult TB, childhood TB has a paucibacillary nature. Childhood TB is then thought to be noninfectious and neglected. Therefore, the management is not prioritized in the TB control programs. Moreover, the symptoms and radiological features may be absent or nonspecific [4]. In addition, compared with adult TB, children TB also have a higher proportion of extrapulmonary form, and the corresponding clinical presentations overlap with other childhood conditions [5]. In fact, except the PTB, the notification of extrapulmonary TB (EPTB) is not mandatory in China, under the Law of Infectious Diseases Control. Therefore, the epidemiological trends in forms of childhood TB remain unclear.

This study is aimed at investigating the prevalence of TB disease and epidemiological trends of TB forms among children in China. The present study may improve the understanding of clinical spectrum of childhood TB and would be helpful to tailor the policy to fit the current situation.

## 2. Materials and Methods

### 2.1. Ethics

The retrospective study was conducted at Shandong Provincial Chest Hospital (SPCH), one of the largest referral TB hospitals in China. This study was approved by the Ethics Committees of SPCH, and the investigations were carried out in accordance with the Declaration of Helsinki. Because of the de-identified data and retrospective nature of the study, written informed consent was waived by the Ethics Committees of SPCH.

### 2.2. Patients

Between January 2007 and September 2020, consecutive children aged ≤ 15 years who were admitted the SPCH for TB were included in the study. Data, including demographic information and underlying diseases, were collected from medical records. Childhood TB is diagnosed based on the combination of symptoms, radiography, tuberculin skin test (or T-SPOT.TB), microbiological examination, biopsy, and response to anti-TB therapy. Cases are categorized and reported according to the anatomical site of TB disease. Pulmonary TB was defined as isolation of TB strains in sputum culture and/or abnormal radiographic findings by chest X-rays and/or chest computed tomography (CT). Extrapulmonary TB was defined as TB with nonpulmonary presentations, such as pleural, lymph nodes, meninges, abdomen, genitourinary system, and musculoskeletal.

Tuberculin skin test was performed using Mantoux method and peripheral blood samples were collected for T-SPOT.TB assay (Oxford Immunotec, Oxford, United Kingdom). Microbiological assays, such as acid-fast bacilli smear (auramine O staining method), TB RT-PCR (IS6110, Daan, Guangzhou, China), and mycobacterial culture (Löwenstein-Jensen media), were used for evaluating suspected childhood TB patients. The biopsy diagnosis of TB was based on the findings for epithelioid cells, multinucleated giant cells, or caseous necrosis [6].

### 2.3. Statistical Analysis

SPSS version 16.0 (SPSS Inc., Chicago, IL) was used for statistical analysis. Patient characteristics were summarized using frequency, mean, and standard deviation measures. To analyze the epidemiological trends in the proportion of each form of TB disease, a linear-by-linear association was used, and a *P* value of <0.05 was considered to indicate that a significant change had occurred in the proportion of TB disease over the studied period.

## 3. Results

During the fourteen-year study period, a total of 1577 children patients were enrolled, including 954 boys (60.5%) and 623 girls (39.5%), with a mean age of 9.26 ± 5.18 years (range: 1 month to 15 years). Sixty-four (4.1%) were found by the radiographic screening (*n* = 61) and the purified protein derivative (PPD) test (*n* = 3). Among the studied patients, 810 (51.4%) patients have PTB, 1137 (72.1%) have EPTB, 372 (23.6%) have the both conditions, and another 765 (48.5%) EPTB cases presented in isolated form. In addition, 228 (14.5%) patients had TB contact history, and 65 (4.1%) were diagnosed as disseminated TB. Five hundred and eighty patients were tested with T-SPOT.TB and the T SPOT-TB assay showed a positive result in 409 (70.5%) patients.

In terms of EPTB, the most common sites were pleura (*n* = 458, 29.0%), followed by the lymph node (*n* = 374, 23.7%), central nervous system (*n* = 149, 9.4%), bone (*n* = 121, 7.7%; 70 cases with spinal involvement), peritoneum (*n* = 57, 3.6%), skin (*n* = 9, 0.6%), urinary system (*n* = 7, 0.4%), pericardium (*n* = 5, 0.3%), intestinal systems (*n* = 5, 0.3%), and so on (Table 1).

The epidemiological trends in forms of childhood TB were further analyzed. During the past decade, the proportion of PTB cases has increased (*P* < 0.05). Similar trends were observed in pleural TB (*P* < 0.01) and tuberculous lymphadenitis (*P* < 0.05).

Meanwhile, we found no significant trend in the proportions of other forms of TB disease, such as EPTB (*P* > 0.05), tuberculous meningitis (*P* > 0.05), endobronchial TB (*P* > 0.05), and disseminated TB (*P* > 0.05). In addition, no significant trend occurred in terms of TB contact (*P* > 0.05).

## 4. Discussion

TB control activities require epidemiological information of TB and improve the control strategies and service delivery systems. In the study, we detailed the epidemiologic trend of childhood TB in a provincial referral TB hospital. Our data showed that pleural TB (29.0%) is the predominant form of EPTB among children, followed by tuberculous lymphadenitis (23.7%), and other forms of TB disease. In addition, during the past decade, the proportions of PTB, pleural TB, and tuberculous lymphadenitis have increased, respectively. To our knowledge, this study was the largest cohort from China over the last decades.

Currently, TB remains one of the ten major causes of mortality among children. However, the actual TB burden in children remains uncertain [7, 8]. According to the Chinese CDC report, the average annual incidence of PTB in children was reported at 2.44/10^5^ [2]. Compared with other countries, the incidence was lower than that of some countries, such as Cambodia and India [1], but still higher than the average global level [9]. Nevertheless, the actual incidence in China would be obviously higher than that reported by the CDC data. This is because that a high proportion of isolated EPTB, as high as 50% in our study, was observed in Chinese childhood TB. In addition, the notification of EPTB is not mandatory according to the Chinese law, and this would underestimate the number of EPTB cases significantly. Considering the challenge of surveillance, estimating the prevalence of TB infection may be useful to assess the prevalence of active TB in children. Recently, the proportion of TB infection in children in China was estimated by Dodd et al. and found it to be 6% [9]. Then, the number of newly diagnosed childhood TB cases was expected at approximately 50000 per annum.

Several studies were conducted to address the distribution of the forms of childhood TB. For example, in Spain, the proportion of EPTB in childhood TB was reported at 17.7%, and the most frequent form of TB disease was tuberculous lymphadenitis (34.5%) [10]. In another study from Pakistan, it was found that 77% of TB disease were pulmonary and 23% were EPTB [11]. Similar results for a low proportion of EPTB among children were reported in Colombia (26%) [12], Ghana (17.4%) [13], and Uganda (20%) [14]. Nevertheless, our study found that a high proportion (about 50%) of isolated EPTB was observed in Chinese childhood TB. We suggest two possible reasons for the disparity in the proportion of EPTB compared with reports from these mentioned countries. First, a high proportion of left axillary lymph node TB was reported in China, and most of them were caused by BCG vaccination [15]. Second, as most of childhood EPTB diagnosed clinically in the study, a high amount of EPTB in children may be caused by overdiagnosis. In addition, likewise, a high proportion of EPTB was also reported in a low-prevalence setting (39.3%) [16]. This was also consisted with data from other studies, such as in Ethiopia (49.5%) [17], Congo (56.1%) [18], and Beijing, China (54%) [19].

In our study, pleural TB and lymph node TB were the most two frequent TB presentations in Chinese children. Other studies also investigated the proportion of EPTB between different countries. For instance, tuberculous lymphadenitis is the most frequent form of EPTB in United States (40%), Netherlands (39%), and United Kingdom (37%) [20–22], whereas pleural TB is the most common form in China (Tianjin, 66%), Romania (58%), and Poland (36%) [23, 24]. In another study from Beijing, China, bone TB (41.1%) and pleural TB (26.0%) were the most two frequent forms of EPTB [25]. These results demonstrated a significant difference between different studies, and this may be explained by geographic region and age.

During the past years, we observed an increasing trend in the proportion of PTB among childhood TB. In fact, during the past years, the prevalence of PTB in China has declined [1]. As known, PTB is responsible for sustaining the disease transmission chain, and the incidence of childhood TB reflects the transmission level of *M. tuberculosis* within a community [26, 27]. This increase may be explained by that (1) the incidence of EPTB declined more rapidly than PTB and (2) EPTB mainly results from reactivation of a previous pulmonary infection [28]. Due to the improvement of the economic status, the reactivation of EPTB was significantly delayed. In a previous study, we found that the proportion of PTB showed a decreasing trend recently [29]. The difference in the proportion of PTB may be caused by an increasing isolation rate of TB strain from EPTB patients, because of the development of TB culture method.

An increasing trend was observed in the proportion of tuberculous lymphadenitis among childhood TB. In a previous study, BCG vaccination was regarded as the most common cause of tuberculous lymphadenitis in children [15]. In the current study, the left lymph node TB comprised about 10% of the total TB patients. Therefore, the incidence of tuberculous lymphadenitis, which mainly caused by BCG vaccination, is stable, and this may be responsible for the increasing trend.

An increasing trend during the past decade was also seen in pleural TB. Similar increase was observed in a recent study conducted by Pang et al. [25]. A previous study demonstrated that pleural TB differs from other forms of EPTB and exhibits the highest clustering rate of all forms of TB, suggesting that pleural TB clustering could be considered as an indicator of recent transmission [25, 30]. Therefore, the increasing trend in pleural TB is thought to be driven by primary transmission. This key point was in agreement with the trend in PTB.

There were some limitations in this study. First, the study was based on a single center analysis and the results may not reflect the whole population of China and generalize to other regions. Second, the TB was diagnosed clinically and might be overdiagnosed; the results should be interpreted cautiously. Third, PPD skin test was considered an important diagnostic tool for TB diseases. However, cross-reaction with atypical mycobacteria and with BCG in vaccinated individuals would limit the use and may have an unfavorable impact on the TB diagnosis. Fourth, data were collected from a provincial referral TB hospital, patients in the hospital tend to have more serious forms of TB disease, such as disseminated TB and tuberculous meningitis. Hence, these study participants may be not representative of childhood TB patients in China.

## 5. Conclusions

Our study helps to understand the prevalence and epidemiological trends of the proportions of TB disease in children in China. Pleural TB and tuberculous lymphadenitis are the most frequent forms of EPTB among children. During the past decade, the proportions of PTB, pleural TB, and tuberculous lymphadenitis showed an increasing trend. Therefore, our findings would be useful in highlighting the burden of TB in children and in developing prevention strategies to protect children from TB infection.

## Figures and Tables

**Table 1:** Distribution of various forms of childhood tuberculosis in Shandong, China, 2006–2019.

Year	Number (*n*)	Pulmonary TB (%)	Extrapulmonary TB (%)	Pleural TB (%)	Tuberculous lymphadenitis (%)	Tuberculous meningitis (%)	Endobronchial TB (%)	Disseminated TB (%)	TB contact history (%)
2006	81	54.30%	63.00%	23.50%	14.80%	8.60%	3.70%	3.70%	4.90%
2007	114	36.00%	73.70%	19.30%	22.80%	11.40%	0.00%	3.50%	11.40%
2008	104	45.20%	61.50%	22.10%	25.00%	5.80%	0.00%	2.90%	16.30%
2009	143	37.10%	75.50%	27.30%	33.60%	8.40%	2.80%	4.20%	11.90%
2010	117	45.30%	72.60%	27.40%	26.50%	14.50%	2.60%	4.30%	13.70%
2011	115	60.90%	76.50%	28.70%	25.20%	13.00%	4.30%	3.50%	18.30%
2012	120	63.30%	67.50%	22.50%	16.70%	11.70%	4.20%	10.00%	21.70%
2013	138	50.00%	79.70%	26.10%	31.20%	12.30%	6.50%	5.10%	11.60%
2014	106	55.70%	72.60%	39.60%	18.90%	8.50%	10.40%	4.70%	14.20%
2015	143	40.60%	82.50%	39.90%	27.30%	6.30%	4.20%	2.10%	14.00%
2016	108	60.20%	65.70%	30.60%	15.70%	10.20%	7.40%	3.70%	12.00%
2017	92	65.20%	68.50%	37.00%	15.20%	3.30%	7.60%	2.20%	13.00%
2018	118	58.50%	65.30%	28.80%	24.60%	6.80%	5.10%	1.70%	16.90%
2019	78	59.00%	76.90%	34.60%	25.60%	10.30%	7.70%	6.40%	23.10%
Total	1577	51.40%	72.10%	29.00%	23.70%	9.40%	4.60%	4.10%	14.50%

## Data Availability

The data used to support the findings of this study are available from the corresponding author upon request.
